# Charles Bonnet Syndrome: Case series

**DOI:** 10.1590/S1980-57642009DN30100012

**Published:** 2009

**Authors:** Sonia Maria Dozzi Brucki, Leonel Tadao Takada, Ricardo Nitrini

**Affiliations:** 1 Department of Psychobiology of Federal University of São Paulo, Brazil.; 2 Cognitive and Behavioural Neurology Unity - Hospital das Clínicas - University of São Paulo, SP, Brazil.; 3 Behavioral and Cognitive Neurology Unit, Department of Neurology, and Cognitive Disorders Reference Center (CEREDIC). Hospital das Clínicas of the University of São Paulo, School of Medicine, São Paulo, SP, Brazil.

**Keywords:** Charles Bonnet syndrome, visual hallucinations, visual loss

## Abstract

Since its first description in 1760, Charles Bonnet syndrome (CBS) has been reported in many studies. The main characteristics are visual hallucinations, preserved awareness of unreal visions, and absence of psychotic symptoms. CBS can occur with lesions located anywhere along the central visual pathway, from the eye to the calcarine fissure.

**Objective:**

To describe patients with CBS and carry out a review of the literature.

**Methods:**

Six patients with visual hallucinations were evaluated in an outpatient memory clinic between 2001 and 2008, and their clinical characteristics recorded.

**Results:**

Four patients were female, and the mean age was 74.5±16.9 years. Three patients had visual loss secondary to eye disease and three due to cerebral lesions. The visions consisted of animals, persons, moving objects, bizarre creatures or colored forms, and were considered disturbing by five patients. Five patients received treatment, and only three reported partial benefit from the therapy. Complete recovery was not seen in any of the subjects.

**Conclusions:**

CBS is relatively rare and its recognition is important to avoid misdiagnoses with psychiatric or dementing illnesses.

## Abstract (pt)

Desde sua descrição em 1760, a Síndrome de Charles Bonnet (SCB) tem sido relatada em vários estudos. Suas características centrais são: alucinações visuais, consciência preservada sobre as alucinações e ausência de sintomas psicóticos. SCB pode ocorrer com lesões desde o olho até o córtex calcarino.

**Objetivo:**

Descrever pacientes com SCB e fornecer revisão da literatura.

**Métodos:**

Seis pacientes com alucinações visuais foram avaliados em um ambulatório de memória no período de 2001 a 2008 e suas características foram descritas.

**Resultados:**

Quatro pacientes eram do sexo feminino, e a idade média foi de 74,5±16,9 anos. Três dos pacientes apresentavam perda visual secundária a lesões oculares e três devido a lesões cerebrais levando a comprometimento visual. O conteúdo das visões consistia de animais, pessoas, objetos em movimento, criaturas bizarras ou formas coloridas. Cinco pacientes receberam tratamento, e apenas três relataram benefício parcial. Melhora completa não foi observada.

**Conclusões:**

SCB é relativamente rara e seu reconhecimento é importante para evitar erros diagnósticos com doenças psiquiátricas ou quadros demenciais.

Charles Bonnet Syndrome (CBS) is characterized by the presence of complex visual hallucinations, frequently associated to visual loss where patients are conscious of the fictitious nature of their hallucinations, and do not present psychotic symptoms.^1^ The disorder was termed CBS in 1967 by de Morsier.^2^

The first report was of Bonnet’s grandfather, who suffered from corneal degeneration and complex visual hallucinations of humanlike figures, birds, and buildings, yet manifested no cognitive or psychiatric disorders (Figure 1).^3^ A wide range of hallucination types have subsequently been reported^4^ and hallucinations have been described in patients with lesions located anywhere from the eye to the calcarine fissure (see below).

**Figure 1:**
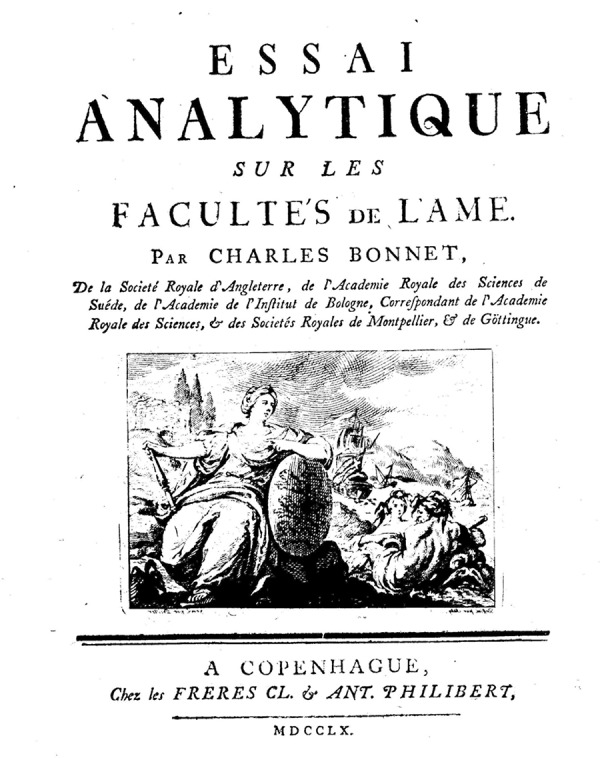
Cover of Charles Bonnet's report.

The prevalence of CBS varies in the literature, with rates ranging from 0.4 to 15%.^5-11^ More recently published studies have reported a lower prevalence of approximately 1% in Asia (0.5% in Japan).^12^ In contrast, prevalence of CBS was 17.5% in 200 elderly with visual impairment in Australia,^13^ and 27.5% in patients with age-related macular degeneration in the United Kingdom.^14^ This syndrome has also been described in a few cases of children with vision loss.^15^ Its prevalence probably underestimated, due to low disclosure by the patients, and owing to various medical conditions associated with the syndrome, as well as to the lack of knowledge about this condition among physicians.

Our aim is to describe a series of six patients with visual impairment and CBS that were evaluated by our group.

## Methods

Patients with visual hallucinations referred for neurologic evaluation in an outpatient memory clinic from 2001 to 2008 (Cognitive and Behavioral Neurology Unit from the Hospital das Clínicas – University of São Paulo School of Medicine) or at one of the author’s private practice (R.N.) and diagnosed with CBS (using the previously reported definition^1^) had their medical records reviewed. During the initial evaluation, besides recording of their demographical, clinical and radiological characteristics, patients were assessed to rule out cognitive impairment using scales including the Mini-Mental State Examination (MMSE).^16^ The patients were subjected to other cognitive tests if deemed necessary.

## Results

The data obtained on the six patients are described in Table 1. Four patients were female, and the mean age (±standard deviation) was 74.5(±16.90) years. Three patients had visual loss due to ocular disease, and three others presented cerebral lesions causing optic chiasmatic compression. All patients had significant visual loss, and developed vivid and bizarre hallucinations including animals, persons, objects or horrid and distorted images. Only one patient did not feel disturbed by the visions. Five patients received oral medications, which included acetylcholinesterase inhibitors, antidepressants and antipsychotics (alone, in combination, or subsequently tried). Although partial benefit was seen in three individuals, complete response was not seen in any of the cases.

**Table 1:** Patient characteristics with Charles Bonnet syndrome.

Case	1	2*	3	4	5	6
Gender	Male	Female	Female	Female	Male	Female
Age	90	83	68	50	63	93
Cause of visual loss	Ocular disease	Macular degeneration	Optic chiasmatic compression	Optic chiasmatic compression	Optic chiasmatic compression	Macular degeneration
Visual acuity	N/A	0.1	Light perception	Count fingers at 2 meters	Amaurosis	Left eye: null; Right eye: 20/400
Frequency	Daily	Almost daily	Daily	Daily	Daily	Daily
Length of symptoms	2 years	1.5 year	3 years	6 years	5 years	1 year
Burden	Moderate	Not disturbed	Mild	Moderate	Mild	Yes
MMSE	26	28	22	23	23	27
Brain imaging study	N/A	N/A	Right frontal encephalomalacia, suprasellar tumor with compression of optical chiasma	Bilateral frontal gliosis	Suprasellar tumor with chiasmatic compression, Right frontal cephalomalacia	Brain CT: normal
Treatment	No	Donepezil	Rivastigmine + Sertraline	Olanzapine	Donepezil + sertraline	Galantamine; sertraline; risperidone; haloperidol
Response to treatment	N/A	No benefit	Partial benefit	Partial benefit	Partial benefit	No benefit
Type of hallucination	Land division, Asian army, mountains, children	Horse wagon	Distorted faces, birds transforming into hats, pigeons with dog faces, and a persistent fluctuating pilaster	Tiny creatures, black mice, snakes, little elephants bears, and monsters	Tiny creatures, fantastic beings, some green grass scenes, branches of herbs	Women dressed in purple clothes; several identical short men with hat; yellow and Black dots drawing a net over his home walls

* Patient 2 has been previously reported 41. N/A, Not available; MMSE, Mini-mental state examination.

In some cases, the hallucinations presented in an unusual manner. Patient 2 reported seeing a horse wagon moving towards her always while being in the front passenger seat of a car. Patient 5 reported visions of landscapes, with green grass and blue sky, associated with a peaceful sensation. Singularly, he was able to voluntarily change unpleasant visions (tiny creatures crawling on his food, snakes, monsters) with these pleasant hallucinations, providing him some degree of relief.

## Discussion

Some risk factors for developing CBS have been described by many authors: visual impairment, cerebral damage, cognitive deficits, social isolation, sensory deprivation, and aging.^17^ Most studies have shown age to be associated with CBS; where, among 500 low-vision patients the syndrome was significantly associated with an age of over 64 years, occurring in around 3% of patients aged 18 to 49 years and 15% in older elderly (75 to 84 years’ old).^7^ Teunisse et al. compared elderly subjects with loss of vision and CBS to those without hallucinations, and found that loneliness and low extraversion were predictors for developing CBS.^18^ In this case series, the mean age was of 74.5±16.9 years, and was in-line with the findings of other reports.^5,9,13,19^

Complex visual hallucinations have been reported following a range of different conditions, and the syndrome stems from a variety of lesions at all levels of the visual system. The most frequent cause is age-related macular degeneration and its treatment (Table 2). In our case series, fifty per cent of our patients demonstrated visual impairment secondary to ocular disease and another half to cerebral lesions of visual pathways. Although visual impairment is not mandatory for the diagnosis of CBS, most authors report a strong association between the two.^17^ All cases in the present series had significant visual disturbance.

**Table 2:** Diseases associated to Charles Bonnet syndrome.

Associated disease	Reference
Macular degeneration	Vukicevic & Fitzmaurice^13^; Khan et al.^14^; Cortizo et al.^41^
Choroidal neovascularization	Brown & Murphy^6^
Treatment for macular degeneration	Meyer et al.^42^
Enucleation of the eye	Tan et al.^43^
Glaucoma	Nesher et al.^11^; Tan et al.^44^
Central retinal artery occlusion	Tan et al.^44^
Optic neuritis, multiple sclerosis	Alao & Hanrahan^34^
Lesions of optic radiation	Freiman et al.^23^
Chiasmal and pituitary lesions	Lepore et al.^45^
Stroke in medial occipital lobe	Cole^46^
Resection of occipital lobe	Choi et al.^47^
Suprasellar meningioma	McNamara et al.^48^
Temporal arteritis, giant cell arteritis	Razavi et al.^49^

Due to the small sample of patients, we cannot draw conclusions regarding gender preponderance in this study. In a review, Menon et al.^17^ found divergent results concerning gender and CBS among various studies. The frequency of the hallucinations is usually reported as occurring daily or weekly.^13,19^ Patients may also report continuous hallucinations or episodic hallucinations with longer intervals.^17^ In five of our patients, the hallucinations occurred daily.

Although previously considered a condition with “pleasant or neutral” symptoms,^1^ later reports have indicated that CBS can be a cause of emotional burden to patients^6,8,10^. Santhouse et al.^19^ found that in a group of 34 patients, the hallucinations generated an emotional response in 50%, and in half of these, the experiences were unpleasant. Vukicevic & Fitzmaurice^13^ reported that the syndrome caused moderate or severe stress in 16 out of 35 patients. In our group, five patients reported a burden associated with the hallucinations. Although this burden was referred to as mild or moderate in most cases, it highlights the importance of correct diagnosis of CBS (reassurance of the sanity of the patient has positive effects)^7^ and of the decision on whether to treat (or at least attempt to treat) the symptoms or otherwise.

Ffytche & Howard^4^ devised hallucination classification into eight categories: tessellopsia (overlapping patterns, repeated geometry); hyperchromatopsia (vivid and bright colors); prosopometamorphopsia (facial distortions, misshapen and mutilated heads); dendropsia (branching forms); perseveration (persistence of details in another scene); illusory visual spread; polyopia (many equal forms); micro/macropsia. The most common patterns in their patients were tessellopsia in 37% and abnormalities of size in 42% of patients, and of these 58% reported micropsia. Among our patients, we identified descriptions matching some of these categories, such as: tessellopsia (“black dots drawing a net over his home walls”), hyperchromatopsia (“women dressed in purple clothes”) polyopia (as in “several identical short men with hats”), micropsia (e.g. “little elephants”) and prosopometamorphopsia (e.g. “distorted faces”). Some patients reported landscapes, animals and/or objects, which could have been seen sometime in the past by them and thus could represent perseveration or long-term pallinopsia.^4^

Particular types of hallucinations are related to some areas of the brain, where the experiences of patients with CBS are associated with activity in extra-striate cortex.^4,19^ Santhouse et al. using fMRI found that, hallucinations involving faces were localized in superior temporal sulcus; objects and scenes in the ventral occipito-temporal cortex, for example.^19^ The notion that these hallucinations could be a release phenomenon provoked by unusual decreased input was described by Cogan, in 1973.^20^ By using single photon emission computed tomography in five patients with CBS secondary to eye disease, Adachi et al. observed that all patients had hyperperfusion in the lateral temporal cortex, striatum and thalamus, and presumed that excessive cortical compensation in these areas could precipitate the syndrome;^21^ using the same methodology (fMRI), increased activity in ventral extrastriate visual cortex was observed.^19^ Burke suggested that hyperactivity in any area will evoke the imagery that is coded by that area.^22^ The hypothesis of deafferentation seems a reasonable cause of these hallucinations, as they are generated in visual association areas.^23^ Cone photoreceptor loss, as in macular degeneration, promotes retino-thalamic-deafferentation, which leads to functional deafferentation of extrastriate cortex.^24^ Burke suggested that hallucinations result from deafferentation of visual structures in the brain, or from the effective silencing of the principal afferents to these structures.^22^ The hypothesis can include two streams of information: one from the periphery to the centre and another in the opposite direction. As the flow from the periphery to the centre diminishes, the contrary flow rises.^25^

Serotonergic activity is related to visual pathways and probably linked to genesis of hallucinations. Serotonin levels are lower in the sensory visual deprived cortex.^26^ Visual information converging in the geniculate nucleus lateral to the visual cortex is modulated by serotoninergic projections from the brainstem.^27^ Acetylcholine is another neurotransmitter involved in visual hallucinations, and concentrated in the visual thalamic nuclei and visual cortex.^28^

The syndrome has an unpredictable outcome, hallucinatory episodes last from seconds to days, and duration of CBS may extend to years. In our cases, some therapeutic options were tried, with partial or no success. As positive outcomes are common with the passing of time, partial responses could be due to spontaneous recovery.

Treatments have been used in reports of single cases or case series in the literature. No controlled clinical trials have been published to date. Reports refer to spontaneous regression, and positive results in some cases with pharmacologic treatment. The majority of studies involve the use of antipsychotic or anticonvulsant drugs. Outcomes are variable, probably due to patient heterogeneity. In particular, the therapeutic response may diverge according to anatomical lesion, albeit ocular or in the brain.

- Anticonvulsants- carbamazepine;^29^ valproate;^30^ gabapentin^31^
- Haloperidol^32^
- Atypical neuroleptics: risperidona,^33^ olanzapine^34^
- Selective serotonin reuptake inhibitors : venlafaxine, citalopram^35^
- Mirtazapine^36^
- Anticholinesterasic drugs: donepezil^37^
- 5-HT3 antagonist – cisapride^38^

Pilsin et al.^39^ have raised the question over whether CBS could be a sign of the initial stages of a dementing illness, having found that patients with CBS performed worse in neuropsychological testing than controls. However, in this study, eight out of fifteen patients had no insight of the illusory nature of their hallucinations, and according to the most frequently accepted definitions,^1^ patients should be diagnosed with CBS only if they are conscious of the unrealistic nature of their visions. There are reports of patients initially diagnosed with CBS – with or without mild cognitive impairment – that later developed Alzheimer’s disease.^40^ The association of CBS and dementia should not lead to the conclusion that CBS is a risk factor for the development of dementia, as advanced age is a risk factor for both conditions while this association (CBS and dementia) is only rarely reported. It is indeed necessary that patients manifesting visual hallucinations, even with concomitant visual impairment, should undergo a thorough evaluation to exclude underlying cognitive impairment. Long-term follow-up is also important, so that initial signs of cognitive deterioration can be detected, should they appear.

There are a number of limitations of this study. Firstly, information on imaging studies was not available in two cases. It is advisable to rule out the presence of structural abnormalities that could explain the visual hallucinations; however, in the cases reported, the absence of other focal neurological signs and accompanying symptoms on follow-up made the possibility of structural causes less likely. We also possessed no information on the influence of the symptoms of CBS on daily life activities, which could substantiate the impact of the symptoms and thus aid in management decisions (as discussed above). It should be noted however that evaluating the impact on daily life activities in visually impaired patients can be problematic.

Summing up, akin to other reported cases of CBS, our patients’ visions contained vivid and colored pattern, a mixture of images, scenes, abnormal sizes, and the subjects showed preserved insight regarding these unrealistic visions. None of the patients responded well to treatment, and among those with some positive response this outcome may not have been due to treatment, but instead to spontaneous remission or fluctuating course. Despite the paucity of treatment options, awareness and recognition of CBS is of the utmost importance to avoid misdiagnoses with dementing or psychiatric illnesses and to offer patients reassurance regarding the integrity of their mental status.
